# Dr. Clifton G. Smith

**Published:** 1899-11

**Authors:** 


### OBITUARY.

Dr. Clifton G. Smith, University of Buffalo, 1887, died at Akron, O., October 2, 1899, aged 35 years.

